# All silicon MIR super absorber using fractal metasurfaces

**DOI:** 10.1038/s41598-023-42723-9

**Published:** 2023-09-20

**Authors:** Alaa M. Ali, AbdelRahman M. Ghanim, Muhammad Othman, Mohamed A. Swillam

**Affiliations:** 1 Department of Physics, School of Sciences and Engineering, The American University in Cairo, New Cairo, 11835 Egypt; 2 Department of Physics, Faculty of Science, Ain Shams University, Cairo, 11566 Egypt

**Keywords:** Nanoscience and technology, Optics and photonics

## Abstract

Perfect absorbers can be used in photodetectors, thermal imaging, microbolometers, and thermal photovoltaic solar energy conversions. The spectrum of Mid-infrared (MIR) wavelengths offers numerous advantages across a wide range of applications. In this work, we propose a fractal MIR broadband absorber which is composed of three layers: metal, dielectric, and metal (MDM), with the metal being considered as n-type doped silicon (D-Si) and the dielectric is silicon carbide (SiC). The architectural design was derived from the Sierpinski carpet fractal, and different building blocks were simulated to attain optimal absorption. The 3D finite element method (FEM) approach using COMSOL Multiphysics software is used to obtain numerical results. The suggested fractal absorber exhibits high absorption enhancement for MIR in the range between 3 and 9 µm. D-Si exhibits superior performance compared to metals in energy harvesting applications that utilize plasmonics at the mid-infrared range. Typically, semiconductors exhibit rougher surfaces than noble metals, resulting in lower scattering losses. Moreover, silicon presents various advantages, including compatibility with complementary metal–oxide–semiconductor (CMOS) and simple manufacturing through conventional silicon fabrication methods. In addition, the utilization of doped silicon material in the mid-IR region facilitates the development of microscale integrated plasmonic devices.

## Introduction

Solar energy, combined with light-trapping technologies, has enormous potential to replace fossil fuels as a clean, secure, and sustainable energy source^1^. Broadband optical absorbers for energy harvesting applications have attracted great research interest in a variety of applications, including photothermal, solar thermophotovoltaics (STPV), photochemical, solar steam production, and catalytic reactions. By employing plasmonic structures, electromagnetic energy can be localized to very small volumes, facilitating effective photon-plasmon conversion at subwavelength scales. Due to their strong optical performance from nanoparticles to nanostructures, plasmonic absorbers have recently been constructed to achieve broadband absorption^2,3^. Light may be controlled and focused in the active layers by carefully constructing these metallic-dielectric nanostructures^4,5^. Broadband plasmonic absorbers with wide-angle incidence, polarization independence, and full absorption across a broad wavelength range are desirable. A substantial amount of research has been conducted to enhance the performance of absorbers^6^.

Plasmonic infrared (IR) absorbers can be utilized in various fields of study. However, achieving a light absorber that has several resonances over a broad spectrum of wavelengths is a complex task. Using structures with multiple resonators to couple various absorption peaks is one approach; however, these structures have a large influence due to the incorporation of multiple components^7^. In addition, other structures with high absorption have been proposed based on metallic plasmonics^8^, Silicon nanowires^9^, and porous silicon^10^; however, they are unstable, costly, and thick.

Implementing fractal metamaterials is one of the approaches used to create broadband absorbers. Metamaterials are periodic structures composed of micro/nanostructural elements of sub-wavelength scale. These structures increase the diffraction limit of conventional optical materials, resulting in optical phenomena such as negative refraction; consequently, metamaterials can be regarded as ideal lenses that improve the interaction between matter and light^11^. In addition, metamaterials are utilized for chiral imaging^12^, cloaking^13^, and concentrators^14^. Moreover, metamaterials are widely used for broadband IR absorbers and thermal camouflage applications^15–17^. The fractal metamaterial technology builds metamaterials using fractal structure. Fractal structures are influenced by natural architectures such as corals, tree branches, and snowflakes; their forming cells and the entire structure exhibit a similar geometry. Multiple resonances enhance the wideband absorption of plasmonic fractal antennas compared to conventional antennas^11^. Fractal plasmonics may involve various configurations, including dendritic^18^, triangular^19,20^, and circular structures^21^, for achieving absorbance in the Terahertz, visible, and infrared regions.

Due to their dependence on metals such as silver or gold, the majority of fractal plasmonics operate in the near-infrared or visible spectrum. Gold and silver are common plasmonic materials in the visible and near-infrared spectral ranges. To take advantage of plasmonic enhancement in the mid-and long-wave infrared regions, materials with plasma frequencies in this range are essential. Plasma frequencies of common metals exist in the visible and near-infrared regions. On the other hand, the plasma frequency of semiconductors can be modulated by varying the concentration of charge carriers (doping) or by applying potential gating. Consequently, highly doped semiconductors emerge as advantageous alternatives to metals in plasmonics, particularly in the infrared region. Several theoretical and experimental investigations have assessed the efficacy of various semiconductor materials. Due to silicon-incorporated photonic devices such as sub-wavelength interconnects, modulators, and emission sources, silicon has attracted considerable interest among these semiconductors^22,23^. Silicon also enables chip-scale integration in the mid-infrared (MID-IR) band, which has numerous potential applications, such as chemical and biological sensors, imagers, light sources, and spectroscopy. In order to increase absorption in the mid-IR region, doped silicon (D-Si) can be used as an alternative for metals^24,25^. In addition, D-Si will make the fabrication of absorbers compatible with CMOS and cost-effective.

Without the need to design a complex grating structure, we study the absorption in the mid-IR wavelength range using the fractal absorber. In this research, we propose a D-Si-based fractal Sierpinski carpet structure. Previously, this structure was introduced by utilizing metals for visible-range absorption. However, the incorporation of D-Si into our fractal structure increased broadband absorption and produced a plasmonic effect in the mid-IR range of 3 to 9 µm.

## Theoretical study

In order to investigate the optical characteristics and near-field computations of the plasmonic absorber, three-dimensional simulations were executed through the utilization of a commercially accessible Maxwell equations software application (COMSOL Multiphysics)^26^. The module of COMSOL Multiphysics uses the finite element method (FEM) to solve a form of the well-known Maxwell’s equation in terms of *E* and *H* over a specified range of frequencies^27^:

$$i\omega \varepsilon E+\sigma E-\nabla \times H=-J$$

1 $$-i\omega \mu H+\nabla \times E=0$$

where $\varepsilon$ is the electric permittivity, $\mu$ is the magnetic permeability, $\sigma$ the conductivity and *J* is the applied current density. We assume $\mu ={\mu }_{0}>0$ and solve for *E*^28^:

2 $$\nabla \times \left({\mu }_{r}^{-1}\nabla \times {\varvec{E}}\right)-{\omega }^{2}{\epsilon }_{0}{\mu }_{0}\left({\varepsilon }_{r}-j\sigma /\omega {\varepsilon }_{0}\right){\varvec{E}}=0$$

where ${\mu }_{r}$ is the relative permeability, ${\varepsilon }_{r}$ the relative permittivity and the magnetic field *B* can be computed from:

3 $$j\omega B=\nabla \times E$$

In order to solve the partial differential equation (PDE), the formulation of the Galerkin for finite elements is used for solving the equation numerically by discretization of the function into a combination of basis functions^28,29^.

**Figure 1:**
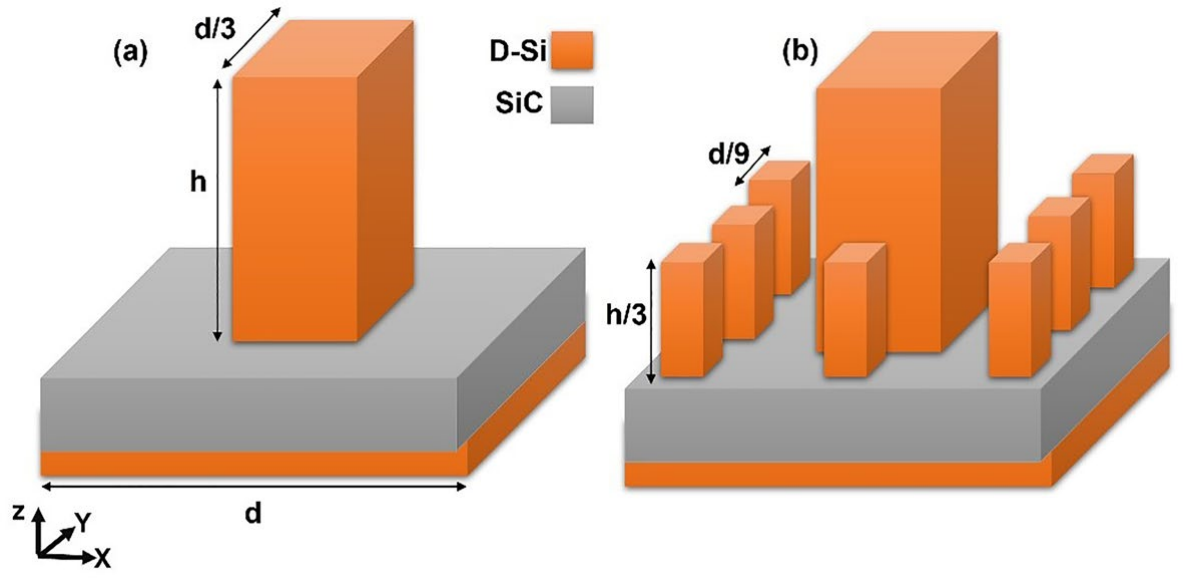
Schematic for Sierpinski carpet fractal structure: (**a**) level-1 and (**b**) level-2.

The optical properties of the optical absorber are initially studied using a plane wave source that is polarized along the *z*-direction with the incident angle *θ* = 45°, as depicted in Fig. 1. The range of wavelengths covered by the incident radiation spectrum is from 1 to 15 μm. The 3D simulation box is enclosed by boundary conditions (BCs) that are periodic, and perfectly matched layers in the *x*, *y*, and *z* directions, respectively. This approach is employed to reduce the computation time. The coefficient of absorption (*A*) can be expressed through the following formula: *A* = 1 − (*R* + *T*) where *R* is the reflection coefficient and *T* is the transmission coefficient.

In this paper, the structure is composed of three layers: metal, dielectric, and metal (MDM); where the metal is substituted with *n*-type D-Si, and the dielectric is Silicon Carbide (SiC). The upper D-Si layer is constructed with a fractal geometry named Sierpinski carpet; it is composed of a square with a side length *d,* the square is divided into a 3 × 3 grid; the central section has a big block with side length *d/3* and height *h*. The other sections have smaller blocks at the center with side lengths d/9 and height *h/3.* The Sierpinski carpet structure was previously proposed in various studies for broadband absorption in the visible region based on metals; however, our proposed structure is the first one to our knowledge to be based on doped silicon for mid-IR region absorption^4,30^. The dielectric layer has a thickness of 180 nm and the below D-Si layer acts as a back reflector with a thickness of 300 nm (Fig. 1). SiC was chosen as a dielectric because it is capable of absorbing a wide range of wavelengths, and it has relatively low density, good thermal and chemical resistance; therefore, it can function under harsh working environments^31^. Floquet periodicity was assumed at the sides of the structure. All the studies were conducted using transverse electric (TE) incident field.

The used D-Si is an *n*-type Si with a Phosphorus doping concentration of 5 × 10^20^ cm^−3^. Drude’s model was used to simulate the D-Si using^32^:

5 $${\varepsilon }_{m}={\varepsilon }_{\infty }-\frac{{\omega }_{p}^{2}}{{\omega }^{2}+i\omega \gamma }$$

where ${\varepsilon }_{m}$ is the complex permittivity of D-Si, $\gamma$ is the collision frequency of value 9.456 × 10^9^ rad/s, ${\varepsilon }_{\infty }$ is the static permittivity of value 11.7, *ω*_*p*_ is the plasma frequency and it is a function of the doping concentration (*N*_*d*_)^32,33^:

6 $${\omega }_{p}= \sqrt{\frac{{N}_{d}{q}^{2}}{{\varepsilon }_{0}{m}^{*}}}$$

where m is the electron rest mass and m* is the effective mass with a value of 0.26 m. *q* is the electron charge. At *N*_*d*_ equals 5 × 10^20^ cm^−3^, the value ${\omega }_{p}$ is 2.474 × 10^15^ rad/s.

In the field of plasmonic-based absorber applications at mid-infrared wavelengths, it has been noticed that doped silicon exhibits better performance compared to metals^24,34^. Typically, semiconductors exhibit smoother surfaces than noble metals, resulting in lower levels of scattering losses. In addition, silicon presents several advantages, including compatibility with complementary CMOS technology and easy fabrication utilizing conventional silicon fabrication methodologies. In addition, the utilization of doped silicon material in the mid-IR region allows the development of microscale integrated plasmonic devices. The combination of these elements enables the production of numerous customary plasmonic devices^35,36^.

## Results and discussions

### Absorber dimensions optimization

**Figure 2:**
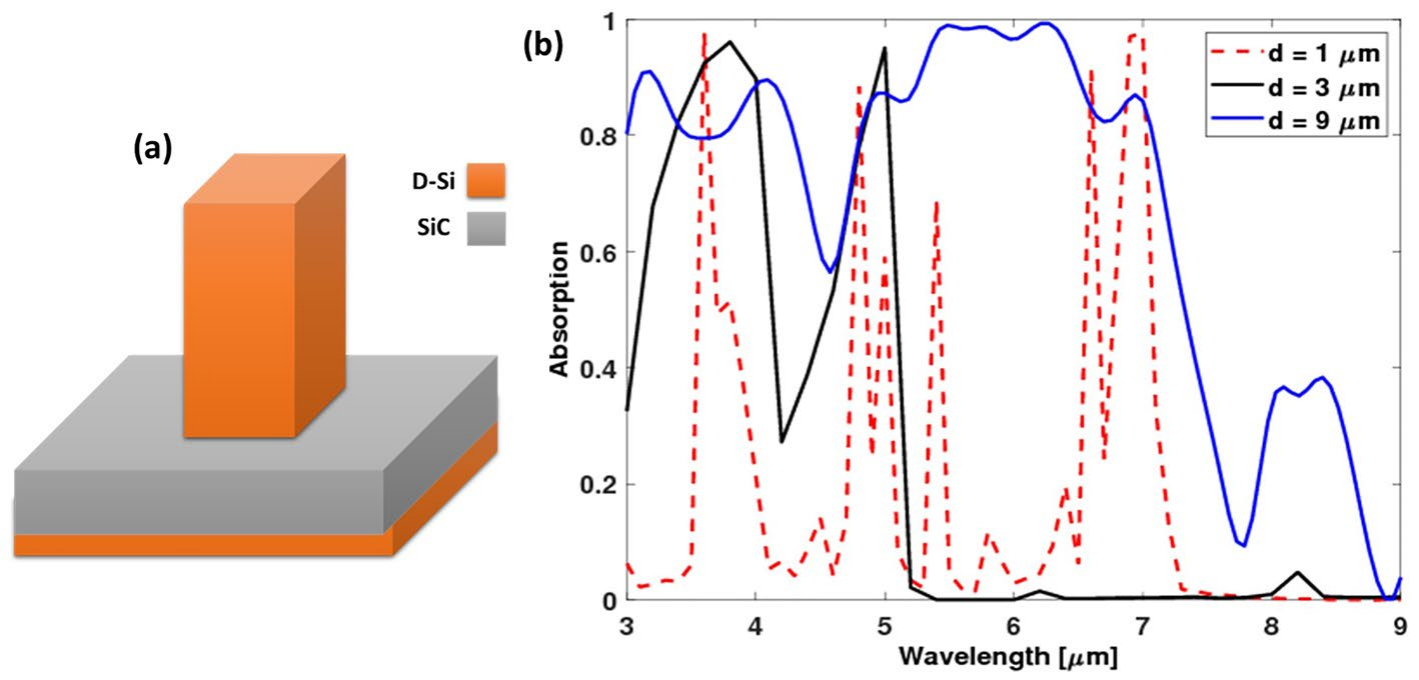
(**a**) Level-1 structure and (**b**) the absorption spectra of different antenna dimensions.

The dimensions of the fractal structure in the level-1 structure (see Fig. 2a) were optimized to give high values of absorption at broadband wavelength. As shown in Fig. 1 the main dimensions are named *d* and *h*, where *h* is always twice *d/3.* Three values of *d* were studied 1, 3, and 9 µm. It has been found that the smaller dimension was unable to absorb the whole range with high intensity; discrete peaks are observed and at the larger wavelengths, the absorption is equal to or near zero. The largest *d* was able to absorb the light with a high percentage in most of the wavelengths in the whole studied range (Fig. 2b). Therefore, the study has proceeded using *d* = 9 µm with square fractal dimensions *d/3* and *h* = 6 µm. The reported enhancement in absorption can be attributed to the interaction between incident light and the plasmonic mode that propagates along the interface of the semiconductor and doped silicon.

### Changing the building block geometry

**Figure 3:**
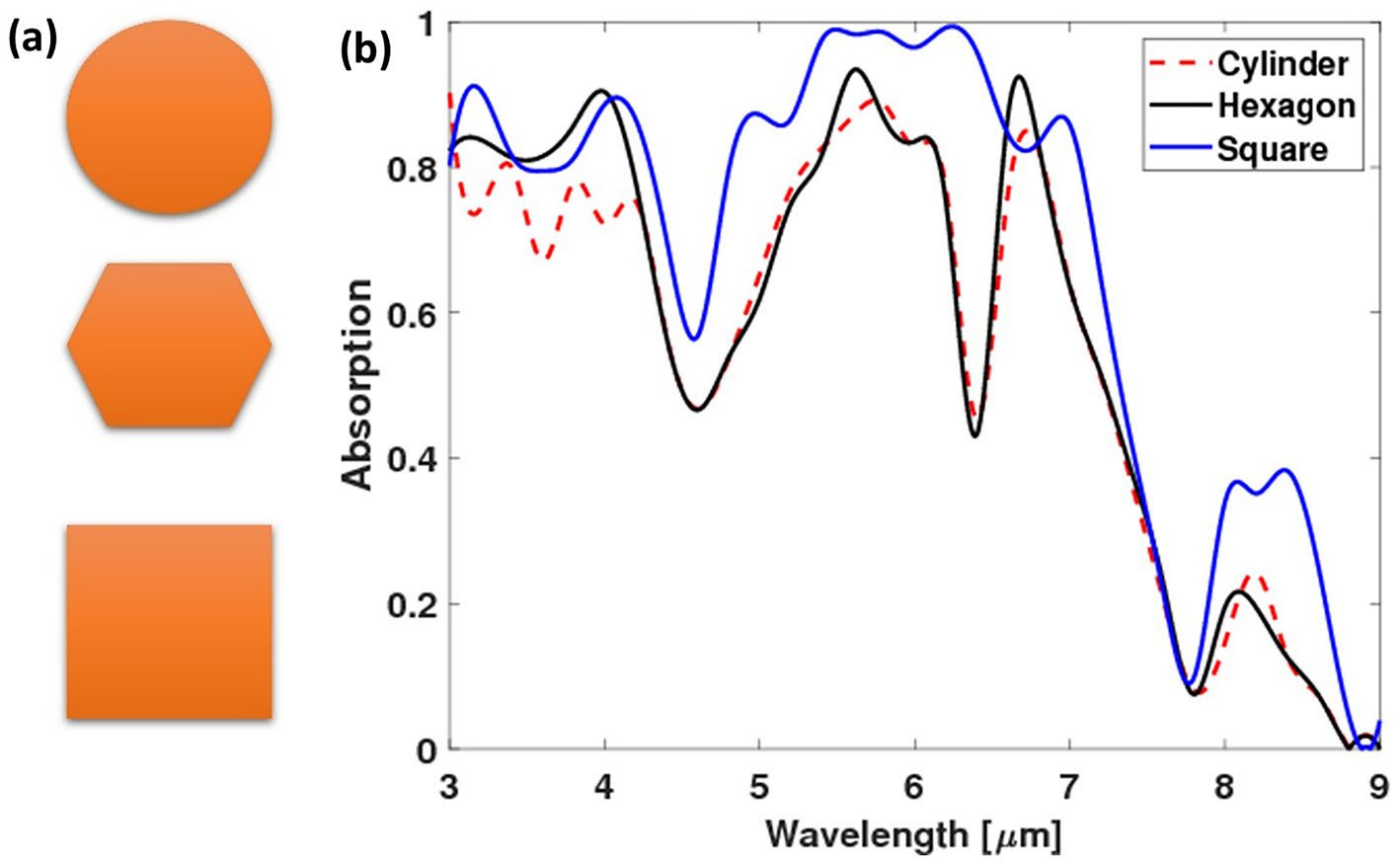
(**a**) Schematic for the three simulated absorbers' geometry, and (**b**) their absorption spectra.

To enhance the absorption of our structure, different building block shapes were simulated where the square cross-section was compared to the circular and hexagonal ones. The building block geometry was changed in the level-1 structure to choose the geometry with the highest absorption. The interface of the cross-section was changed to circular, hexagonal, or square. It is found that the square interface gave the highest absorption compared to the other geometries at most of the wavelengths in the studied range (Fig. 3).

### Fractal structure

**Figure 4:**
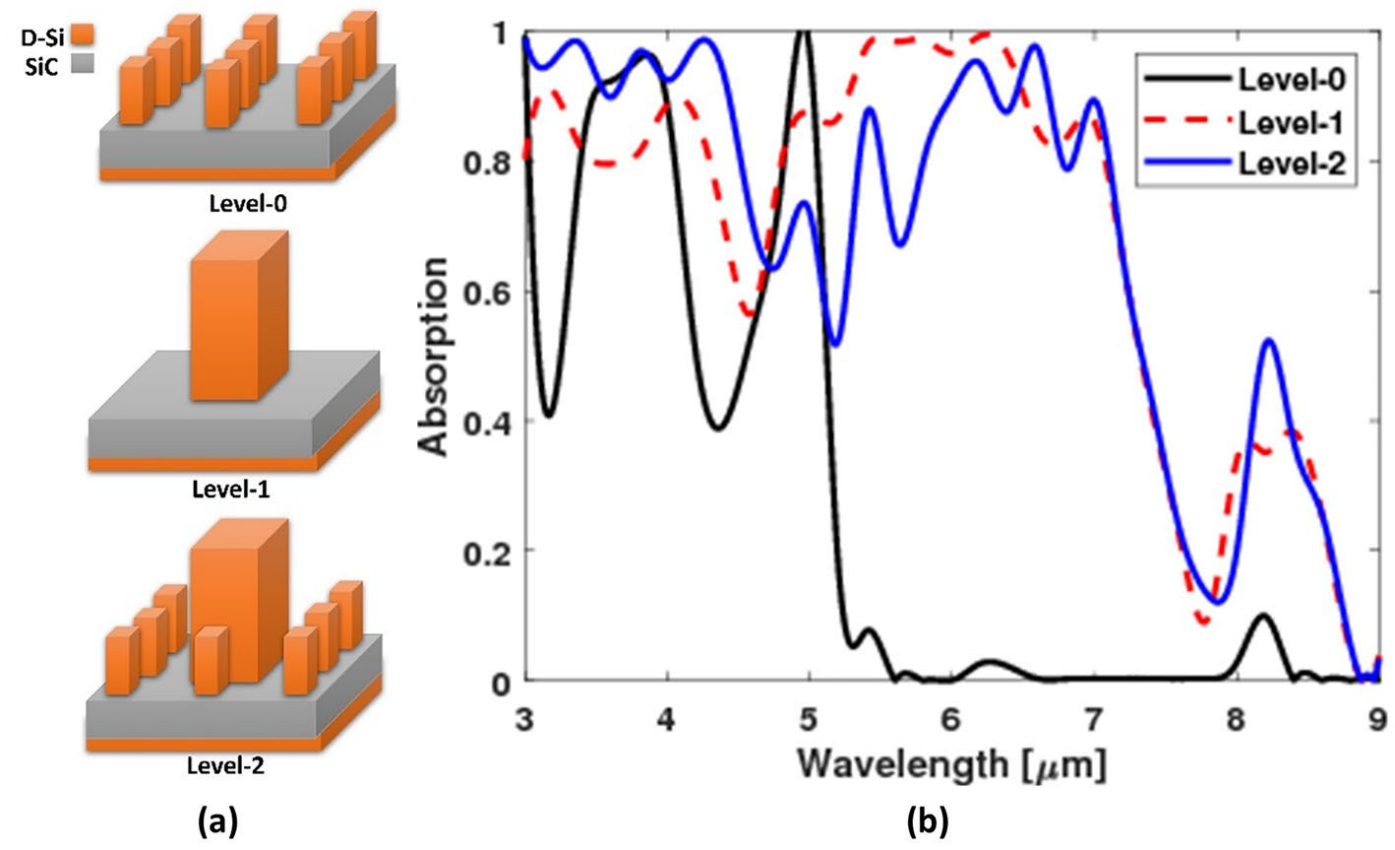
(**a**) Schematic for the three simulated absorbers geometry, and (**b**) their absorptions.

The fractal structure was built for more enhancement of the absorption using the block of the square interface (Fig. 1). The absorption of level-1 and level-2 fractal structures were compared. Also, a structure with the small blocks only (level-0) was simulated (Fig. 4a) to see the effect of the big block and the fractal structure. It is observed that the level-2 fractal structure led to enhancement of the absorption especially at wavelengths smaller than 5 µm and larger than 6.5 µm with increasing the absorption peak at around 8.3 µm as shown in Fig. 4b.

**Figure 5:**
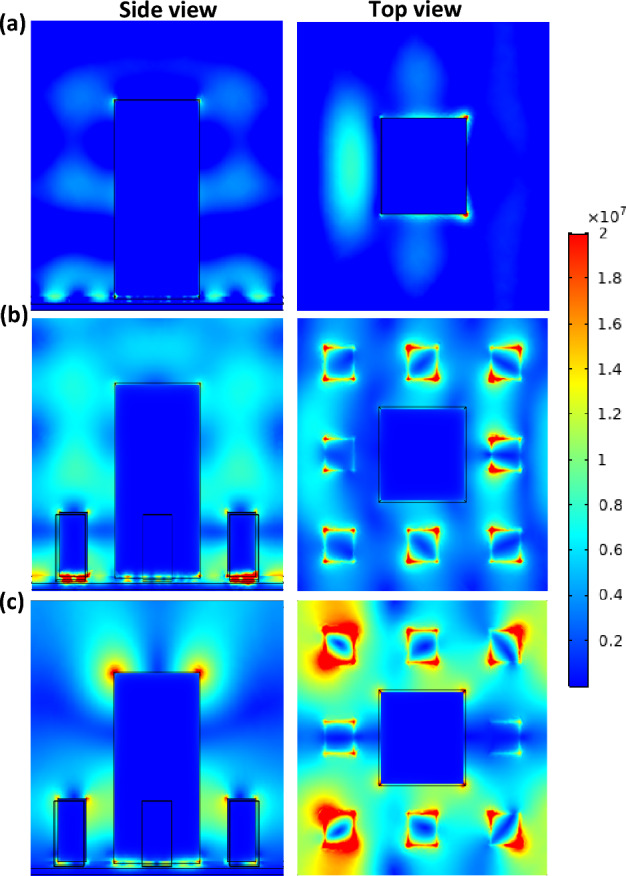
Electric field intensity of (**a**) Level-1 Sierpinski carpet at *λ* = 4.2 µm, (**b**) Level-2 Sierpinski carpet at *λ* = 4.2 µm, and (**c**) Level-2 Sierpinski carpet at *λ* = 6.8 µm.

When looking at the electric field distribution in the level-1 fractal structure at one of the wavelengths of high absorption, it is found that it is concentrated around the square element (Fig. 5a). While in level 2, it is observed two behaviors: one for the low wavelengths such as 4.2 µm, and higher wavelengths such as 6.8 µm. At smaller wavelengths with high absorption, it is found that the electric field is concentrated around the smaller blocks only as shown in Fig. 5b. On the other hand, when the larger wavelengths are absorbed, the electric field is concentrated around both the big block and the smaller ones as can be seen in Fig. 5c. This indicates that incorporating the smaller blocks with the bigger one to form the fractal structure enhances the absorption at a broader range. Moreover, the electric field is trapped between the fractal structure and the SiC layer below.

The high absorption is attributed to multiple electric/magnetic dipolar resonances and the presence of surface plasmon (SP) modes; moreover, the presence of a back-reflector plays an important role in absorption enhancement by concentrating the electric field at the dielectric layer. For the absorption at the lower wavelengths, an intense enhancement of the localized electromagnetic fields has emerged between the two plasmonic layers. This can be attributed to charge accumulation at the blocks which couple to the charge in the back reflector generating strong electric dipole resonances leading to the confinement of an electromagnetic field at the intermediate dielectric spacer^4^. At higher wavelengths, the absorption is attributed to the excitation of SP modes due to the coupling effect of the big square element with the incident field. The same effect happens at the lower wavelengths with the smaller elements. So, the main advantage of SP modes is their tunability by changing the size of the absorber^22,37^.

### Further absorption enhancement by core–shell structure

**Figure 6:**
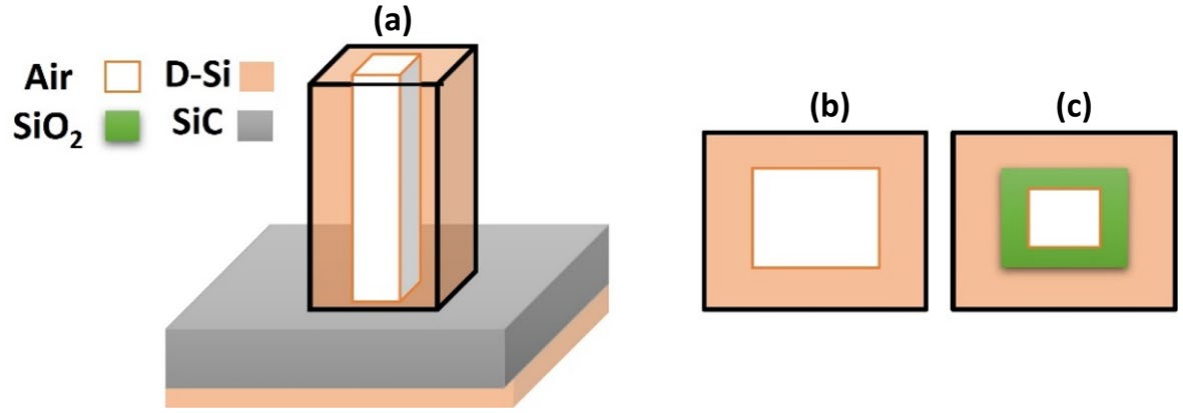
Schematic for the modified structure with an absorber made of metamaterials (level-1): (**a**) side view of fractal absorber with air core, (**b**) top view of a fractal with air core, and (**c**) with air core surrounded by a border of SiO_2_.

**Figure 7:**
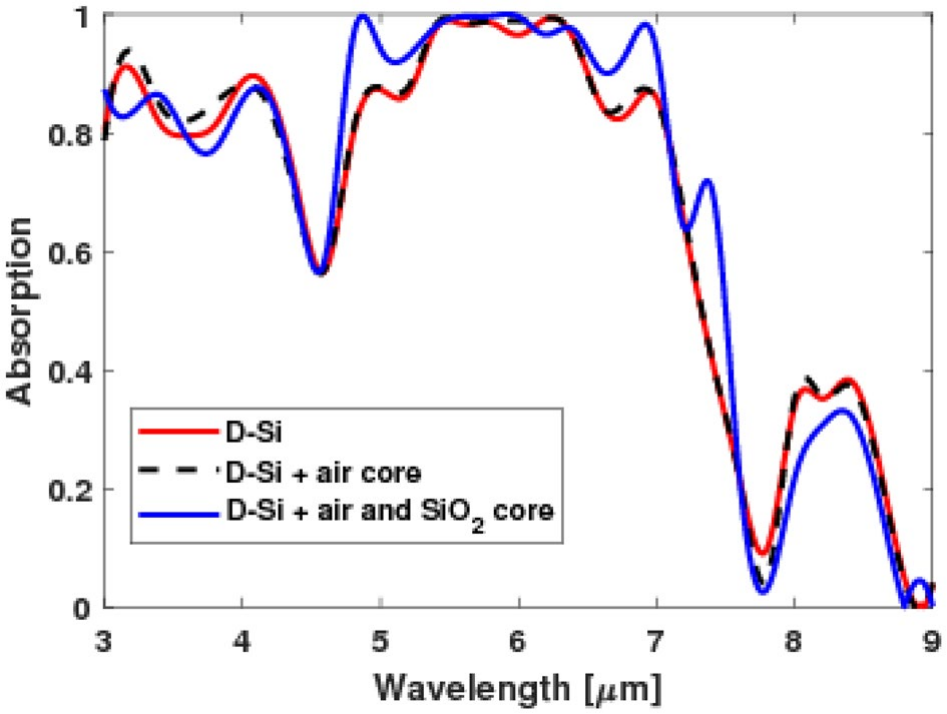
A comparison between the absorption spectra for a fractal structure (level-1) made of D-Si, D-Si with air core, and D-Si with air core surrounded by SiO_2_.

The materials of the fractal in the level-1 structure were modified to have a metastructure too. Level-1 structure was simulated by implementing an air core in the middle of the D-Si with width d/9 (Fig. 6a and b). Moreover, the air core was surrounded by a border of Silicon dioxide (SiO_2_) with a width of d/9 too (Fig. 6c). It was found that the air core led to enhancement of the absorption at the smaller wavelengths. Interestingly, the structure with SiO_2_ led to the highest absorption when compared to the other structures at a broad range between 4.5 and 7.5 µm as shown in Fig. 7.

**Figure 8:**
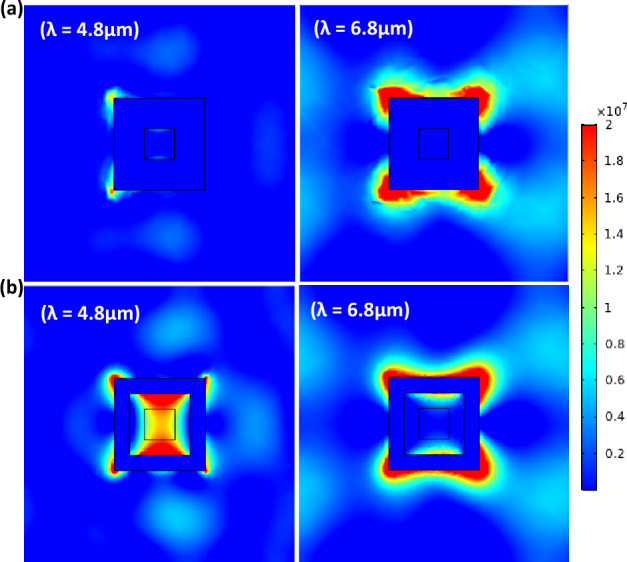
Top view of electric field profile of level-1 (**a**) with an air core and (**b**) air core surrounded by a border of Silicon dioxide.

The electric field distribution in the three different materials structure shows that it appears with a considerable percentage around the level-1 structure with air core (Fig. 8a). In addition, it also appears between the plasmonic and the dielectric layer in the level-1 structure with air and SiO_2_ core (Fig. 8b). This indicates that the metastructure of the fractal led to a generation of more SP modes enhancing the absorption.

**Figure 9:**
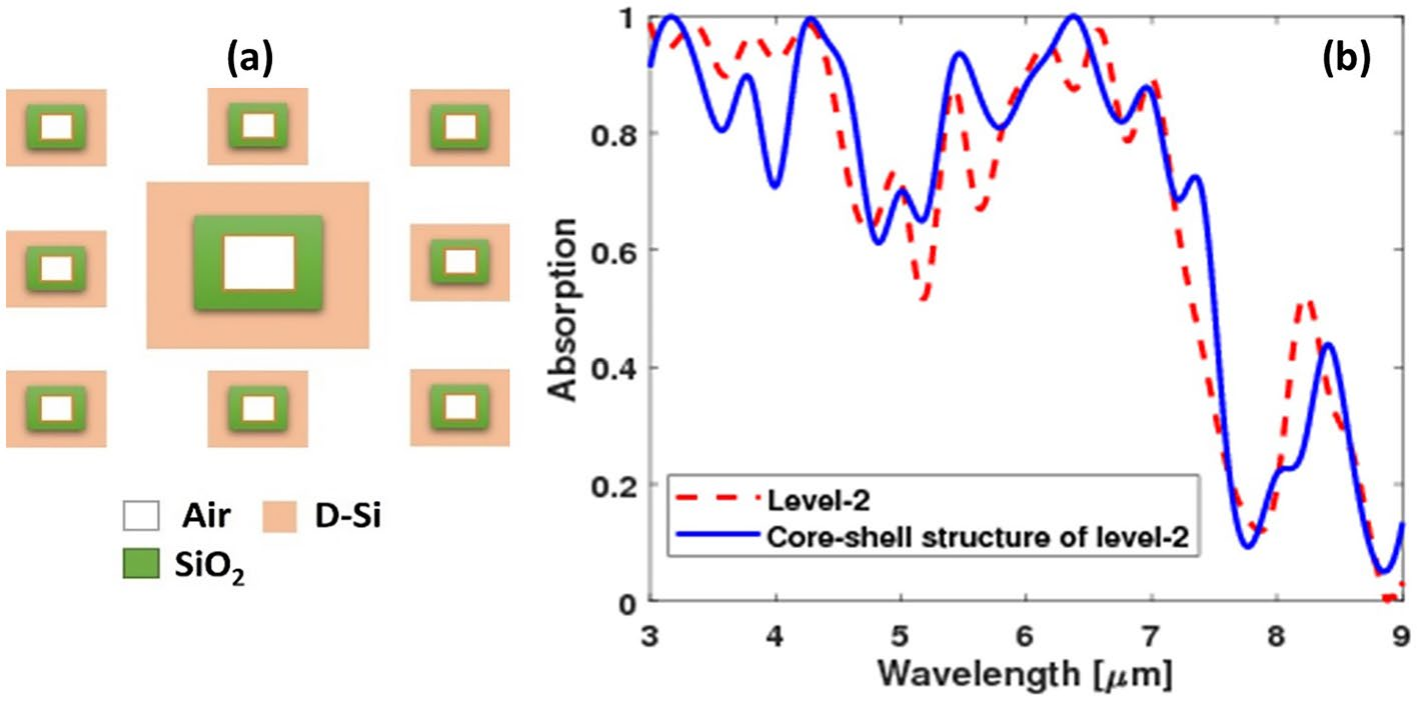
(**a**) Top view of the core–shell structure of level-2 and (**b**) the absorption spectra as a function of wavelength for Level-2 and core–shell structure of level-2.

For further absorption enhancement, the level-2 structure's materials were modified to include the core–shell. The Level-2 structure was simulated by incorporating a wide air core in the center of the D-Si and the air core was surrounded by a Silicon dioxide (SiO_2_) border (Fig. 9a). It was observed that the core–shell structure of level-2 increased the absorption in a wide range between 4.5 and 7.5 µm as shown in Fig. 9b.

**Figure 10:**
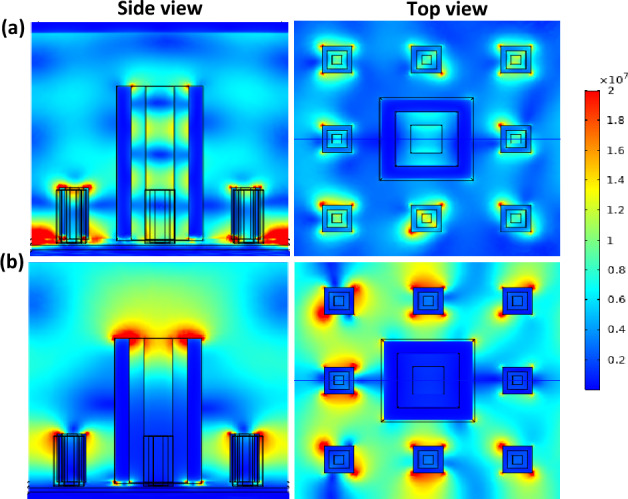
Top view of electric field intensity of the level-2 with air core surrounded by SiO_2_ at (**a**) *λ* = 4.2 µm and (**b**) *λ* = 6.4 µm.

Due to the high density of available photonic states associated with the iso-frequency surface, these concentric tubes can generally maintain an increased number of propagating light modes^22,38^. Increasing the coupling between incident light and modes enables the realization of multiple loss mechanisms at various wavelengths, resulting in broad absorption. In addition, the presence of a periodic array with an air core in the tubes creates a photonic crystal that, depending on the diameter of the air core, provides another type of resonance mechanism. In order to show the excitation of multiple loss mechanisms, Fig. 10a and b depict the electric field intensity |*E*|^2^ profile at discrete wavelengths across the wavelength of interest investigated. This is due to bulk plasmon modes being highly confined to the bulk of the composite material^39^.

Table 1 depicts a comparison between the proposed fractal structure and the previously reported metamaterial absorbers. The results demonstrate that the proposed structure obtains an average absorptivity of greater than 98% in the band of 3–9 μm with a relative absorption bandwidth of 100%, which significantly broadens the perfect absorption band in comparison to previous research. In addition, the use of dielectric materials reduces production costs, allowing for the mass production of absorbers.

**Table 1:** The comparison of the proposed design to the previous absorbers.

Ref.	Region (µm)	Relative bandwidth (%) = 2(*λ*_*Upper*_ − *λ*_*Lower*_)/(*λ*_*Upper*_ + *λ*_*Lower*_)	Resonator material	Material type	Cost
^40^	0.5–1.81	113	W	Metal	Low
^41^	0.48–1.48	102	Au	Metal	High
^42^	0.4–2.0	133	W	Metal	Low
^43^	0.4–1.8	127	Au, Ge	Metal + dielectric	High
^44^	0.3–3.0	164	Fe, Cr	Metal	Low
^45^	7–15	73	Ti	Metal	High
^46^	8–20	86	Mo + Ge_2_Sb_2_Te_5_	Metal + alloy	High
Proposed	3–9	100	D-Si	Dielectric	Low

### Suggested fabrication steps

**Figure 11:**
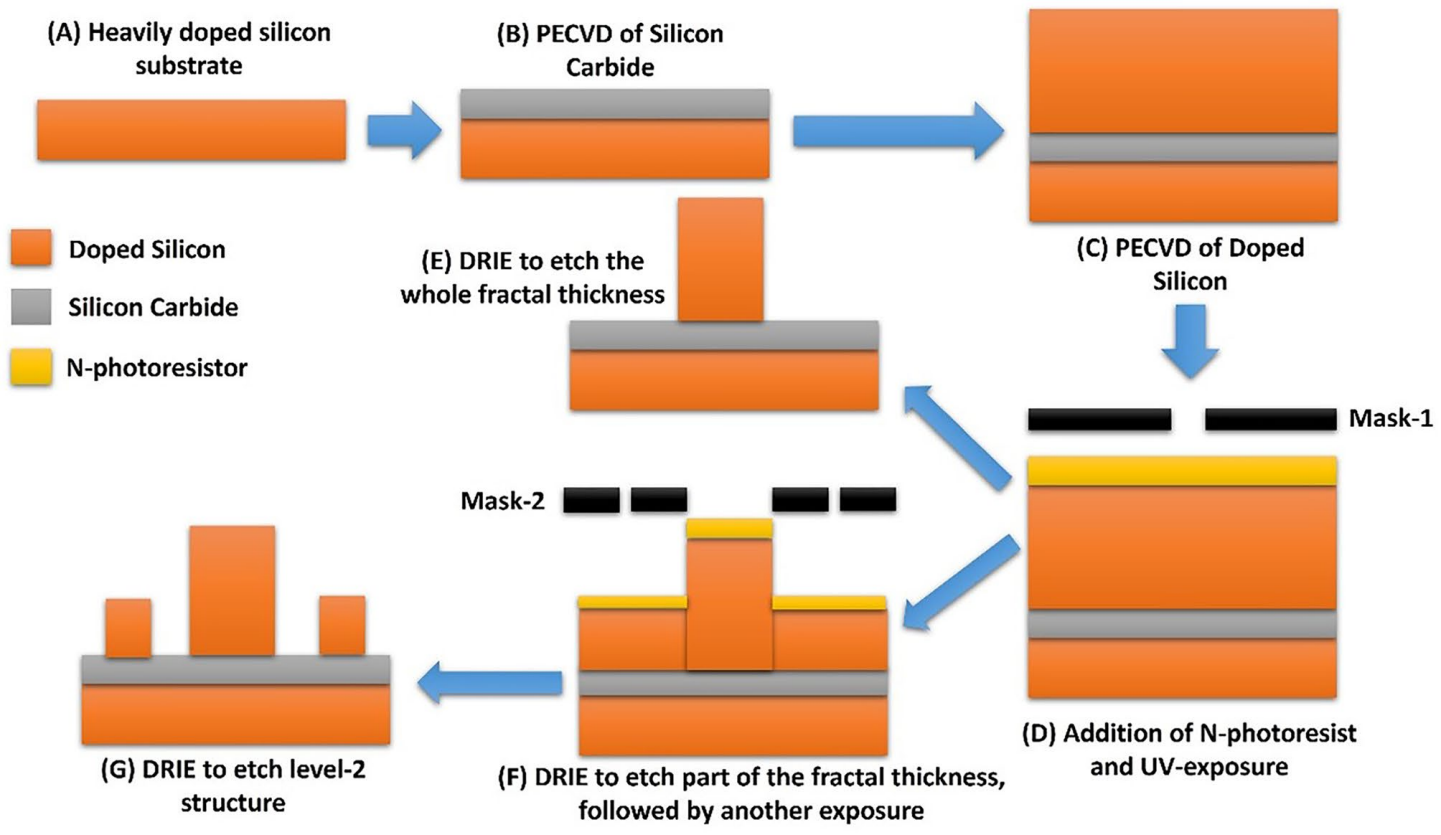
Schematic for Sierpinski carpet level-1 and level-2 fractal structures suggested fabrication steps.

**Figure 12:**
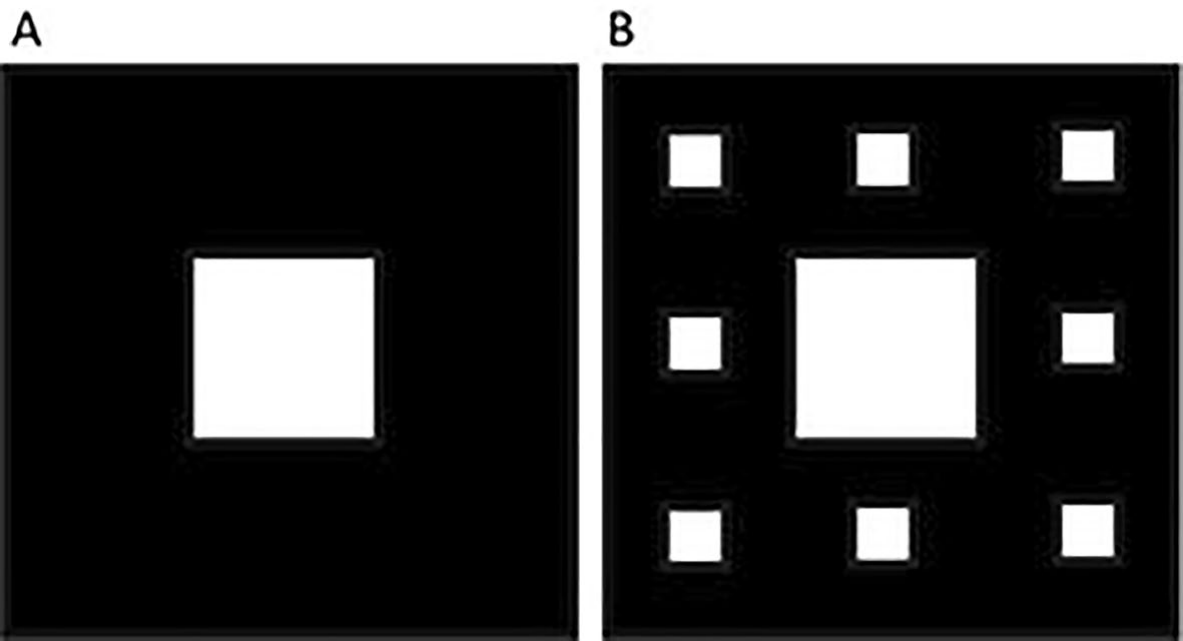
Design of the masks to be used in the fabrication steps. (**A**) Mask 1 and (**B**) Mask 2.

The suggested fabrication process for the Sierpinski carpet structures starts with a heavily doped Si wafer to form the back reflector (Fig. 11A). Then, a layer of SiC is added using Plasma Enhanced Chemical Vapor Deposition (PECVD) (Fig. 11B)^47^. Also, a layer of D-Si is deposited using PECVD (Fig. 11C). Thickness control for each layer should be performed by controlling the process time and temperature. After that, a photolithography step will be required. One mask will be needed to fabricate a level-1 structure (mask 1). Mask 1 will be designed to have a square perforated chrome over quartz glass substrate where the square will have the dimensions of the big square element (Fig. 12A). After UV exposure using this mask and a negative photoresist (N-photoresistor) (Fig. 11D), Deep Reactive Ion Etching (DRIE) will be performed to get the fractal structure (Fig. 11E)^48^. To fabricate the level-2 Sierpinski carpet structure; the lithography and the DRIE steps will be done in two stages. The first stage is using mask 1 followed by DRIE but with less time than in the fabrication of level-1 structure, to only etch around the higher part of the central element (Fig. 11F). Then, another mask will be used (mask 2) where the pattern it has is the Sierpinski carpet of a central big square, and smaller squares around it (Fig. 12B). Another exposure step and DRIE are needed to get the final level-2 Sierpinski carpet structure (Fig. 11G)^49^.

Also, to create the structure with SiO_2_ borders (in Section “Further absorption enhancement by core–shell structure”) SiO_2_ will be deposited using PECVD after the SiC, then photolithography and DRIE will be performed to get a SiO_2_ column with the needed dimensions. Then PECVD of D-Si will be done and the steps in Fig. 11 starting from step (D) will be followed to reach the final level-1 structure with SiO_2_ core. To create an air core inside the fractal, another mask will be needed with a square of dimensions equal to the air core followed by DRIE^50^. Therefore, making the building block fractal with metastructure will make the fabrication steps more complicated.

## Conclusion

In this study, we proposed a fractal Sierpinski carpet structure based on D-Si. The metastructure is composed of three layers: the lowest layer is made of *n*-type D-Si and over it a dielectric layer composed of SiC. The highest layer is also made of a D-Si layer constructed with a Sierpinski carpet fractal structure. The advantage of using D-Si is to get a plasmonic effect at the mid-IR range of 3 to 9 µm using a CMOS-compatible and cost-effective material and fabrication process. The advantage of using fractal metastructure is to enhance the broadband absorption by generating multiple electric/magnetic dipolar resonances in addition to surface plasmon modes. Different studies were conducted to optimize the shape and size of our absorber. Three different shapes of the fractal geometry were simulated, and it was found that the fractal with a square cross-section will lead to higher and wider absorption as compared to the circular and hexagonal ones. Moreover, the level-2 Sierpinski carpet fractal structure was found to enhance the absorption when compared to a level-1 structure or a structure with equally sized fractals due to the generation of different SP modes with different wavelength ranges. Moreover, the existence of dielectric layers between two plasmonic layers led to the generation of electric/magnetic dipolar resonances. For further enhancement of the absorption, the materials of the fractal in the level-1 structure were modified to have a metastructure too. Level-1 structure was simulated by implementing an air core in the middle of the D-Si. Moreover, the air core was surrounded by a border of Silicon dioxide (SiO_2_), and it was found both structures can lead to higher absorptions at different ranges. However, the structure with SiO_2_ led to the highest absorption when compared to the other structures at a broad range between 4.5 and 7.5 µm. Therefore, Using D-Si with our fractal structure led to the enhancement of broadband absorption and gave a plasmonic effect at the mid-IR range. The absorption was higher than 0.9 at most of the studied wavelength range. The suggested fabrication steps were illustrated, as further work is needed to experimentally verify our proposed structure.

## Data Availability

The datasets used and/or analyzed during the current study available from the corresponding author on reasonable request.
